# Detection of closely linked QTLs and candidate genes controlling germination indices in response to drought and salinity stresses in barley

**DOI:** 10.1038/s41598-024-66452-9

**Published:** 2024-07-08

**Authors:** Hossein Sabouri, Zahra Pezeshkian, Fakhtak Taliei, Mahjoubeh Akbari, Borzo Kazerani

**Affiliations:** 1 Department of Plant Production, College of Agriculture Science and Natural Resource, Gonbad Kavous University, Gonbad, Iran; 2 Department of Animal Sciences, Faculty of Agricultural Sciences, University of Guilan, Rasht, Iran; 3 BioGenTAC Inc., Technology Incubator of Agricultural Biotechnology Research Institute of Iran-North Branch (ABRII), Rasht, Iran; 4 Department of Plant Breeding and Biotechnology, Faculty of Plant Production, Gorgan University of Agricultural Sciences and Natural Resources, Gorgan, Iran

**Keywords:** Barley, Candidate genes, Drought, Germination, Salinity, Biotechnology, Genetics, Molecular biology, Plant sciences

## Abstract

The aim of current study was to identify closely linked QTLs and candidate genes related to germination indices under control, salinity and drought conditions in barley. A total of nine (a major), 28 (eight major) and 34 (five major) closely linked QTLs were mapped on the seven chromosomes in response to control, drought and salinity conditions using genome-wide composite interval mapping, respectively. The major QTLs can be used in marker-assisted selection (MAS) projects to increase tolerance to drought and salinity stresses during the germination. Overall, 422 unique candidate genes were associated with most major QTLs. Moreover, gene ontology analysis showed that candidate genes mostly involved in biological process related to signal transduction and response to stimulus in the pathway of resistance to drought and salinity stresses. Also, the protein–protein interaction network was identified 10 genes. Furthermore, 10 genes were associated with receptor-like kinase family. In addition, 16 transcription factors were detected. Three transcription factors including B3, bHLH, and FAR1 had the most encoding genes. Totally, 60 microRNAs were traced to regulate the target genes. Finally, the key genes are a suitable and reliable source for future studies to improve resistance to abiotic stress during the germination of barley.

## Introduction

Barley (*Hordeum vulgare* L., 2n = 2x = 14, HH genome) is the fourth most important cereal crop in the world^1^. This plant has been one of the essential grains in the ancient world, but today it is mostly used as animal feed and malt. It is also sometimes used in the human diet^2^. In recent years, with the increasing intensity of global warming and climate change, abiotic stresses such as drought and salinity have increased, and these factors are among the most important limiting factors for the growth and development of cereals in arid and semi-arid regions such as Iran. Despite the relative tolerance of barley to abiotic stresses, under severe stress conditions, the quantity and quality of barley yield are damaged^3^. Specially, among the stages of plant growth and development, the germination stage is known as one of the most sensitive growth stages under abiotic stress conditions^4^.

Overall, one of the primary and important responses of plants to abiotic stresses such as drought and salinity is the increased concentration of reactive oxygen species (ROSs) in chloroplasts, mitochondria, and peroxisomes. At low concentrations, ROSs play a positive role as signaling molecules in regulating growth and multifactorial stress responses. However, at high concentrations, ROSs cause damage to macromolecules such as proteins, lipids, and nucleic acids. As a result, plants utilize antioxidant defense mechanisms to regulate and reduce ROSs concentrations, thereby regulating the redox state of the cell^5–7^. Moreover, drought and salinity stresses are among the main factors of osmotic stress, which have a detrimental effect on plant growth and crop yield^8^. However, in addition to osmotic stress, salinity stress also damages plants through ionic toxicity. Salinity stress, in two phases including osmotic (rapid) and ionic (slow), damages plants by limiting the growth of young leaves and accelerating the senescence of mature leaves, respectively. However, plants tolerant to salinity stress adapt to saline conditions through three important mechanisms including osmotic stress tolerance, removal of sodium and chloride ions, and tolerance to the accumulation of these ions. In plants tolerant to salinity stress, some gene families such as *HKT* play a role in removing sodium ions from leaves^9^. At the seedling morphology level, salinity stress at various levels reduces the germination percentage (GP), shoot length (SL), root length (RL), shoot fresh weight (SFW) and root fresh weight (RFW)^10^. Additionally, drought stress during the seedling stage decreases GP, plumule length and radicle length^11^.

Today, molecular techniques are used to evaluate the defense mechanisms of plants against biotic and abiotic stresses. Accordingly, to identify quantitative trait loci (QTLs) and marker-trait associations (MTAs) in response to biotic and abiotic stress conditions, QTL mapping and association analysis techniques have been used in biparental and artificial populations, respectively^12–14^. In several studies using the mentioned techniques, the defense mechanism of barley against abiotic stresses has been investigated at the germination stage. For example, in a study, the effect of drought stress on 60 spring barley genotypes was evaluated during the germination and seedling stage, and as a result, 71 QTLs were identified on seven barley chromosomes using the single-marker analysis (SMA) technique^15^. In another study, the effect of salinity stress on 103 doubled haploid (DH) lines of barley was evaluated at the seedling stage, and to prepare a linkage map, 350 diversity arrays technology (DArTs) and 84 simple sequence repeats (SSRs) were used. In total, 6 QTLs were tracked on chromosomes 1H, 3H, and 4H using the multiple-QTL mapping technique^16^. Finally, in a study, the effect of drought and salinity stresses on 143 European winter barley cultivars was investigated at the seedling stage. Also, to perform the genome-wide association study (GWAS) technique, 4855 single-nucleotide polymorphisms (SNPs) were used, and as a result, 28 QTLs were tracked. Additionally, four loci on chromosomes 1H, 5H, and 6H responded to two growth conditions, and based on this, the researchers found that a QTL controlling seedling growth under one abiotic stress condition probably responds to another stress as well^17^. Accordingly, it is likely that some of the responses of plants to drought, salinity, heat, and cold stresses are common, and the reason for this is the destructive effects of recent stresses through the reduction of water potential (osmotic stress)^18^.

Overall, QTLs controlling tolerance to abiotic stresses and the gene network associated with them can provide a better understanding of the improvement and tolerance to abiotic stresses. Also, this kind of information will greatly contribute to a deeper understanding of the molecular and physiological mechanisms of the plant in response to abiotic stresses. Therefore, the primary objective of the present study was to track closely linked QTLs for tolerance to drought and salinity stresses and the candidate genes related to major QTLs in Iranian barley. Moreover, functional analysis and regulatory factors such as microRNAs (miRNAs), transcription factors (TFs) and protein kinases related to these candidate genes were the ultimate goal of the present study. Also, the current study was a first report with the mentioned objectives on Iranian barley.

## Results

### Phenotypic assessment

The germination indices including GP, RL, SL, germination index (GI), germination rate index (GRI), seedling of vigor index (SVI), mean germination time (MGT), seedling weight vigor index (SWVI), seedling length vigor index (SLVI), root length index (RLI), root dry weight index (RDWI), shoot length index (SLI), shoot dry weight index (SDWI), root/shoot ratio by length (R/SL), root/shoot ratio by dry weight (R/SDW), root/shoot ratio by length index (R/SLI) and root/shoot ratio by dry weight index (R/SDWI) were evaluated. Based on this, descriptive statistics for germination indices were calculated. Descriptive statistics including mean and range of germination indices were as central tendency and dispersion indices, respectively. These statistics were provided general information about the center and range of data. Also, Pearson’s skewness and kurtosis tests, as indices of vertical and horizontal symmetry, determined the frequency distribution of data (Supplementary Table 1). Based on the latter test, the phenotypic distribution of data in all germination indices was normal, and as a result, the germination indices had a quantitative nature and the data had a continuous distribution.

### Linkage map construction

The SSR, ISSR, EST, TE, SCoT, CBDP, IRAP, RAPD, ISJ, iPBS, iPBS-iPBS combined, and combined ISSR-iPBS markers were distributed 217, 72, 56, 26, 27, 29, 7, 66, 73, 138, 3 and 5 polymorphic alleles, respectively. Chromosomes 1H (120 markers), 2H (90 markers), 3H (114 markers), 4H (110 markers), 5H (100 markers), 6H (82 markers) and 7H (103 markers) were covered 133.26, 120.01, 169.72, 161.32, 142.87, 144.94 and 172.94 cM of barley genome, respectively (Supplementary Fig. 1).

#### Closely linked QTLs controlling germination indices in control condition

A total of nine closely linked QTLs associated with GP, SL, GI, SLVI and R/SL were mapped on chromosomes 1H, 2H, 4H, 5H and 7H under control condition. qGP-C-1H, explaining 20.37% of phenotypic variation, was identified as a major QTL (Table 1).

**Table 1:** The closely linked QTLs associated with germination indices under control condition in barley.

Trait	QTL	Chr	Pos (cM)	LOD	Additive effect	Left marker	Right marker	R^2^	Allele direction
GP-C	**qGP-C-1H**	1H	45.23	3.11	− 1.36	iPBS2240-C	iPBS2240-C	20.37	Kavir
SL-C	qSL-C-4H	4H	65.37	4.44	0.57	ISSR47-5	ISSR47-5	15.70	Badia
	qSL-C-5H	7H	105.27	3.79	0.58	HVCMA	HVCMA	16.12	Badia
GI-C	qGI-C-2H	2H	30.69	2.65	0.11	ISSR30-2	UMB206	12.00	Badia
SLVI-C	qSLVI-C-4H	4H	129.16	2.54	11.39	ISSR16-4	ISSR16-4	11.05	Badia
R/SL-C	qR/SL-C-1H	1H	16.21	3.28	− 0.09	OPB-01-A	OPB-01-A	6.72	Kavir
	qR/SL-C-2H	2H	18.14	5.00	0.14	ISJ17-B	HvXan	16.28	Badia
	qR/SL-C-5H	5H	136.13	3.11	-0.11	GBM1166	IRAP54-2	9.74	Kavir
	qR/SL-C-7H	7H	14.24	2.89	0.13	AF022725A	ISSR21-1	13.97	Badia

GP-C, germination percentage in control condition; SL-C, shoot length in control condition, GI-C, germination index in control condition; SLVI-C, seedling length vigor index in control condition; R/SL-C, root/shoot ratio by length in control condition. The major QTLs were marked with bold.

#### Closely linked QTLs controlling germination indices in drought stress condition

A total of 28 closely linked QTLs related to RL, SL, GI, SWVI, SLVI, RDWI, SLI, SDWI, R/SL, R/SDW and R/SLI were located on chromosomes 1H, 2H, 3H, 4H, 5H, 6H and 7H under drought stress condition. The coefficients of determination for qRL-D-7H, qSL-D-5H, qGI-D-2H, qSLI-D-1H, qSLI-D-5Hb, qR/SL-D-7H, qR/SDW-D-3H and qR/SLI-D-6H were 20.48%, 20.40%, 31.46%, 21.27%, 20.38%, 24.02%, 21.37% and 2.01%, respectively. As a result, they were introduced as major QTLs (Table 2).

**Table 2:** The closely linked QTLs associated with germination indices under drought stress condition in barley.

Trait	QTL	Chr	Pos (cM)	LOD	Additive effect	Left marker	Right marker	R^2^	Allele direction
RL-D	qRL-D-2H	2H	6.98	4.44	− 0.80	iPBS2078-C	iPBS2078-C	18.57	Badia
	**qRL-D-7H**	7H	22.77	3.51	0.84	ISSR48-6	HvSMEi868b	20.48	Kavir
SL-D	qSL-D-1H	1H	62.31	3.43	0.81	iPBS2218-B	CBDP5-D	18.22	Kavir
	**qSL-D-5H**	5H	30.54	4.06	− 0.86	HVM30	GBM1426	20.40	Badia
	qSL-D-7H	7H	105.27	3.27	0.60	HVCMA	HVCMA	10.11	Kavir
GI-D	**qGI-D-2H**	2H	108.49	4.33	0.17	GBMS202	GBM1462	31.46	Kavir
	qGI-D-5H	5H	132.36	2.99	− 0.09	iPBS2221-2	iPBS2221-2	8.89	Badia
SWVI-D	qSWVI-D-1H	1H	124.09	3.11	0.14	OPD-05-B	OPD-05-B	7.69	Kavir
	qSWVI-D-2H	2H	18.97	2.69	− 0.16	ISJ17-B	HvXan	9.90	Badia
	qSWVI-D-4H	4H	49.34	2.51	0.19	UMB711	Bmag0490	14.06	Kavir
	qSWVI-D-6H	6H	79.90	3.20	0.20	ISJ11-A	ISJ4-D	14.27	Kavir
	qSWVI-D-7H	7H	105.27	2.70	0.12	HVCMA	HVCMA	5.48	Kavir
SLVI-D	qSLVI-D-1H	1H	61.74	4.23	11.08	iPBS2218-B	iPBS2218-B	14.12	Kavir
	qSLVI-D-5H	5H	27.66	3.53	− 9.78	HVM30	HVM30	11.01	Badia
	qSLVI-D-7H	7H	105.27	2.98	9.94	HVCMA	HVCMA	11.36	Kavir
RDWI-D	qRDWI-D-5H	5H	47.36	3.17	6.72	iPBS2274-C	iPBS2274-C	13.53	Kavir
	qRDWI-D-7H	7H	169.79	2.62	− 8.01	iPBS2229-B	ISJ24-A	19.21	Badia
SLI-D	**qSLI-D-1H**	1H	62.31	4.16	7.42	iPBS2218-B	CBDP5-D	21.27	Kavir
	qSLI-D-5Ha	5H	0.90	3.13	5.09	ISSR29-1	EBmac0560	10.00	Kavir
	**qSLI-D-5Hb**	5H	29.58	5.02	− 7.27	HVM30	GBM1426	20.38	Badia
SDWI-D	qSDWI-D-1H	1H	62.31	3.05	6.55	iPBS2218-B	CBDP5-D	18.39	Kavir
	qSDWI-D-4H	4H	33.09	2.76	− 3.60	ISSR31-6	ISSR31-6	11.53	Badia
	qSDWI-D-5H	5H	20.07	2.83	− 4.31	iPBS2415-3	iPBS2415-3	7.98	Badia
	qSDWI-D-7H	7H	105.27	4.18	5.93	HVCMA	HVCMA	15.08	Kavir
R/SL-D	**qR/SL-D-7H**	7H	22.77	4.47	0.09	ISSR48-6	HvSMEi868b	24.02	Kavir
R/SDW-D	qR/SDW-D-1H	1H	90.21	3.49	0.06	Bmac0090	Bmac0090	15.47	Kavir
	**qR/SDW-D-3H**	3H	151.55	2.84	− 0.20	ISSR22-1	ISSR31-5	21.37	Badia
R/SLI-D	**qR/SLI-D-6H**	6H	81.18	3.40	− 0.09	iPBS2253-A	iPBS2253-A	20.01	Badia

RL-D, root length in drought stress; SL-D, shoot length in drought stress; GI-D, germination index in drought stress; SWVI-D, seedling weight vigor index in drought stress; SLVI-D, seedling length vigor index in drought stress; RDWI-D, root dry weight index in drought stress; SLI-D, shoot length index in drought stress; SDWI-D, shoot dry weight index in drought stress; R/SL-D, root/shoot ratio by length in drought stress; R/SDW-D, root/shoot ratio by dry weight in drought stress, R/SLI-D, root/shoot ratio by length index in drought stress. The major QTLs were marked with bold.

#### Closely linked QTLs controlling germination indices in salinity stress condition

A total of 34 closely linked QTLs affecting GP, RL, SL, GRI, SVI, MGT, SWVI, RLI, RDWI, SLI, R/SDW and R/SDWI were located on chromosomes 1H, 2H, 3H, 4H, 5H, 6H and 7H under salinity stress. qGP-S-2H, qRL-S-5Ha, qGRI-S-2H, qSWVI-S-2H and qR/SDW-S-4H explained 26.68%, 22.69%, 26.67%, 24.30% and 21.12% of phenotypic variations, respectively. Therefore, these QTLs were recognized as major QTLs (Table 3).

**Table 3:** The closely linked QTLs associated with germination indices under salinity stress condition in barley.

Trait	QTL	Chr	Pos (cM)	LOD	Additive effect	Left marker	Right marker	R^2^	Allele direction
GP-S	**qGP-S-2H**	2H	31.78	3.17	3.79	UMB206	ISSR16-1	26.68	Kavir
RL-S	qRL-S-1H	1H	6.25	3.79	− 1.02	iPBS2401-C	iPBS2401-C	8.47	Badia
	qRL-S-3H	3H	161.27	4.32	1.03	GBM1238	GBM1238	8.71	Kavir
	**qRL-S-5Ha**	5H	60.55	4.79	1.66	HVM30a	B06-D	22.69	Kavir
	qRL-S-5Hb	5H	131.28	3.68	− 1.02	GBM1508	GBM1508	8.53	Badia
	qRL-S-7H	7H	93.25	3.26	− 1.24	SCoT6-A	Bmag0135	12.68	Badia
SL-S	qSL-S-4H	4H	161.32	2.90	− 0.48	EBmac0658	EBmac0658	12.06	Badia
GRI-S	**qGRI-S-2H**	2H	31.78	3.17	6.72	UMB206	ISSR16-1	26.67	Kavir
SVI-S	qSVI-S-1H	1H	61.74	2.67	0.86	iPBS2218-B	iPBS2218-B	11.88	Kavir
MGT-S	qMGT-S-5H	5H	96.74	2.99	0.047	ISSR38-7	ISSR38-7	11.10	Kavir
SWVI-S	**qSWVI-S-2H**	2H	18.97	2.99	− 0.23	ISJ17-B	HvXan	24.30	Badia
RLI-S	qRLI-S-4H	4H	60.97	2.70	− 7.79	ISJ11-B	scssr14079	19.62	Badia
RDWI-S	qRDWI-S-1H	1H	126.42	2.54	− 5.44	CBDP1-A	CBDP1-A	12.87	Badia
	qRDWI-S-5H	5H	61.08	3.19	− 5.25	B06-D	B06-D	12.00	Badia
SLI-S	qSLI-S-1H	1H	27.09	4.50	− 4.35	ISJ10-C	ISJ10-C	11.99	Badia
	qSLI-S-4H	4H	62.18	5.68	− 4.64	ISJ15-B	ISJ15-B	13.65	Badia
	qSLI-S-5H	5H	139.84	4.32	5.43	iPBS2375-B	iPBS2375-B	18.72	Kavir
	qSLI-S-7H	7H	105.27	3.38	3.92	HVCMA	HVCMA	9.74	Kavir
R/SDW-S	qR/SDW-S-1H	1H	2.58	3.45	0.08	iPBS2298-B	iPBS2298-B	10.60	Kavir
	qR/SDW-S-2H	2H	16.40	3.10	0.08	SCoT7-C	ISJ17-B	10.13	Kavir
	qR/SDW-S-3H	3H	18.14	2.96	− 0.08	iPBS2384-B	iPBS2384-B	10.14	Badia
	**qR/SDW-S-4H**	4H	55.70	2.90	0.08	EBmac0906	OPB-04-B	21.12	Kavir
	qR/SDW-S-5H	5H	115.20	2.68	− 0.06	CBDP5-A	CBDP5-A	5.92	Badai
	qR/SDW-S-6H	6H	30.36	2.65	− 0.06	iPBS2380-C	iPBS2380-C	5.24	Badai
R/SDWI-S	qR/SDWI-S-1H	1H	2.58	2.98	− 7.57	iPBS2298-B	iPBS2298-B	6.32	Badia
	qR/SDWI-S-2H	2H	18.14	3.69	− 8.76	ISJ17-B	HvXan	8.46	Badia
	qR/SDWI-S-3Ha	3H	52.37	3.27	4.48	GBM1450	GBM1450	2.22	Kavir
	qR/SDWI-S-3Hb	3H	77.11	4.24	− 5.26	GBM1037	GBM1037	3.05	Badia
	qR/SDWI-S-4H	4H	35.92	5.97	7.31	GBM1350	GBM1350	5.90	Kavir
	qR/SDWI-S-5Ha	5H	115.20	4.52	7.88	CBDP5-A	CBDP5-A	6.84	Kavir
	qR/SDWI-S-5Hb	5H	130.64	5.58	8.56	B15-C	GBM1508	8.08	Kavir
	qR/SDWI-S-7Ha	7H	14.24	2.59	− 9.11	AF022725A	ISSR21-1	9.15	Badia
	qR/SDWI-S-7Hb	7H	41.81	4.64	− 8.34	ISSR29-6	Bmac0144j	7.66	Badia
	qR/SDWI-S-7Hc	7H	105.12	3.01	− 4.24	D15-A	D15-A	1.98	Badia

GP-S, germination percentage in salinity stress; RL-S, root length in salinity stress; SL-S, shoot length in salinity stress; GRI-S, germination rate index in salinity stress; SVI-S, seedling of vigor index in salinity stress; MGT-S, mean germination time in salinity stress; SWVI-S, seedling weight vigor index in salinity stress; RLI-S, root length index in salinity stress; RDWI-S, root dry weight index in salinity stress; SLI-S, shoot length index in salinity stress; R/SDW-S, root/shoot ratio by dry weight in salinity stress; R/SDWI-S, root/shoot ratio by dry weight index in salinity stress. The major QTLs were marked with bold.

### Candidate genes

**Figure 1:**
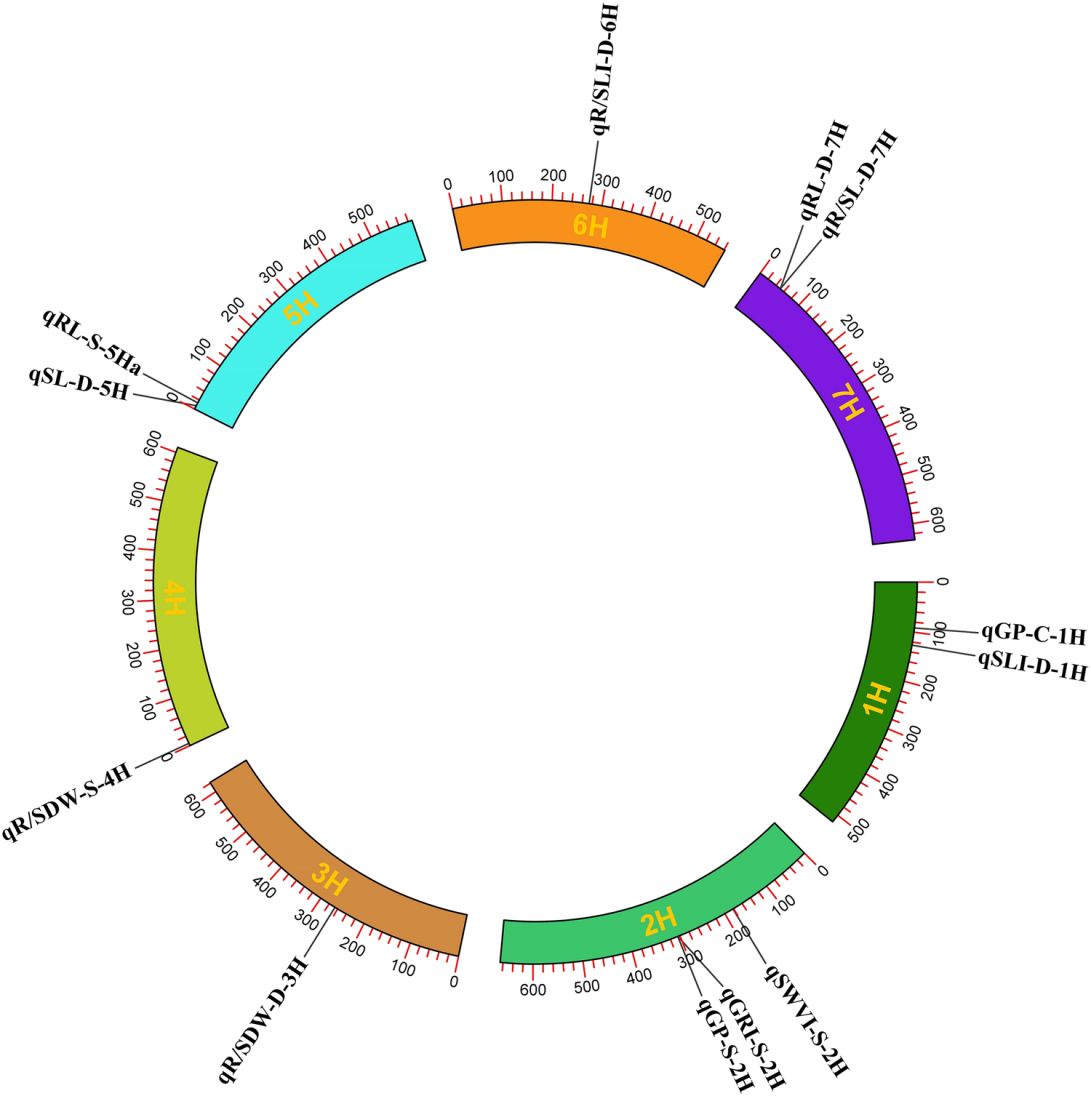
A circular plot represents the names and positions of QTLs on the seven barley chromosomes.

In the present study, 14 major QTLs were identified under different environmental conditions including control (a major QTL), drought (eight major QTLs) and salinity (five major QTLs). Then, the candidate genes of the major QTLs were tracked. However, no significant genes were identified for two QTLs including qGI-D-2H and qSLI-D-5Hb. As a result, the candidate genes were identified for 12 major QTLs including qGP-C-1H, qSLI-D-1H, qSWVI-S-2H, qGRI-S-2H, qGP-S-2H, qR/SDW-D-3H, qR/SDW-S-4H, qSL-D-5H, qRL-S-5Ha, qR/SLI-D-6H, qRL-D-7H and qR/SL-D-7H. Based on this, a total of 501 candidate genes were associated with 12 major QTLs under control, drought and salinity conditions. Totally, 44, 49, 3, 111, 140, 9 and 145 candidate genes were located on chromosomes 1H to 7H, respectively. qR/SDW-S-4H and qSL-D-5H were related to 111 and 91 genes, respectively. Some candidate genes were common among major QTLs. Therefore, only 422 unique candidate genes were identified as final candidate genes and were used for further analysis. A circular plot showed the position of each major QTL on each chromosome (Fig. 1).

### Gene Ontology (GO) analysis

**Figure 2:**
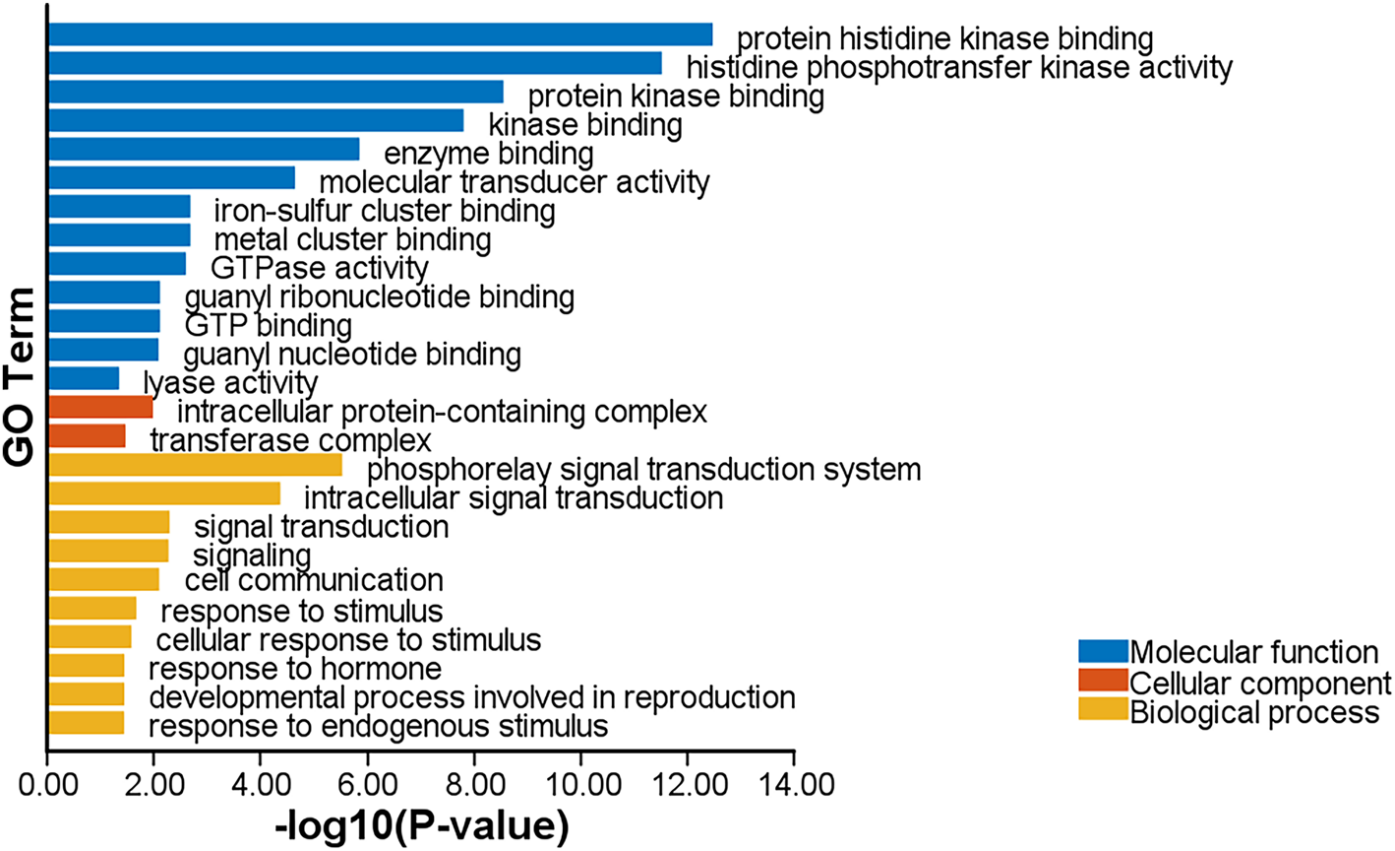
Gene ontology analysis of candidate genes related to 12 major QTLs.

GO analysis was performed for all candidate genes of 12 major QTLs. GO analysis was categorized into three classes including biological process, molecular function, and cellular components. The most important biological processes involved included phosphorelay signal transduction system, intracellular signal transduction, signal transduction, signaling, cell communication, response to stimulus, and cellular response to stimulus. The most important molecular functions are protein histidine kinase binding, histidine phosphotransfer kinase activity, protein kinase binding, kinase binding and enzyme binding. Two main cellular components involved were intracellular protein-containing complex and transferase complex (Fig. 2).

### Protein–Protein Interaction (PPI) network

**Figure 3:**
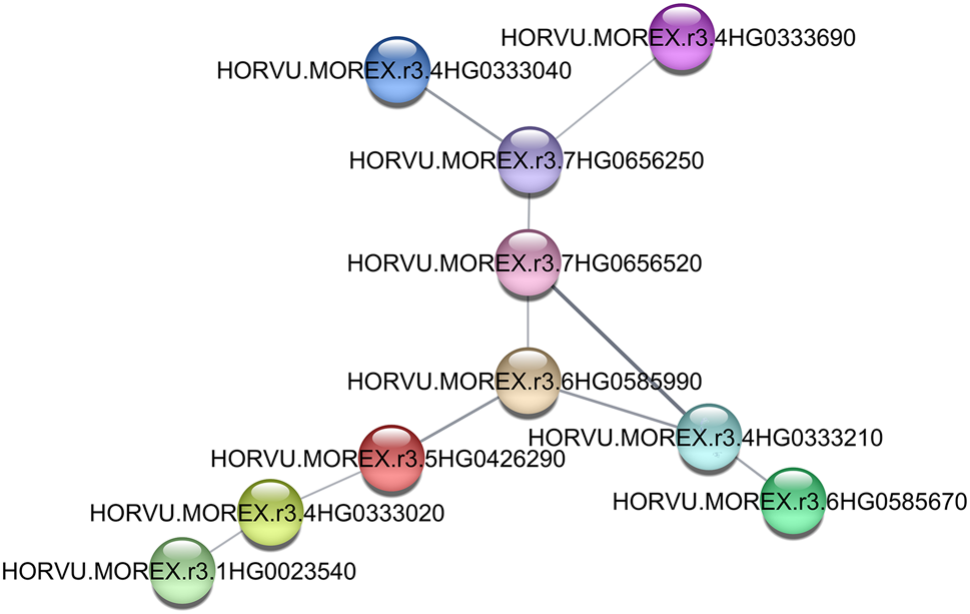
Protein–protein interaction networks of ten genes.

The PPI network of candidate genes was investigated. The information about the protein names of this species in string was not a lot, therefore, a small network was created (Fig. 3). Totally, there are ten genes in this network which related to each other. One of them was HORVU.MOREX.r3.4HG0333690 which is regulatory protein NPR1. NPR1 and WRKY are identified as the master regulators of systemic acquired resistance^19^. Another gene was HORVU.MOREX.r3.7HG0656250 that is involved in ubiquitin-dependent protein catabolic process. The ubiquitin–proteasome system is a key role in regulating protein stability and turnover in plants, especially during adverse environmental conditions like drought, salinity, cold, and nutrient deprivation^20^. Most of these genes were involved in the response to abiotic stress through various pathways and processes such as regulation of DNA-templated transcription, nucleotide-excision repair, etc.

### TFs

**Figure 4:**
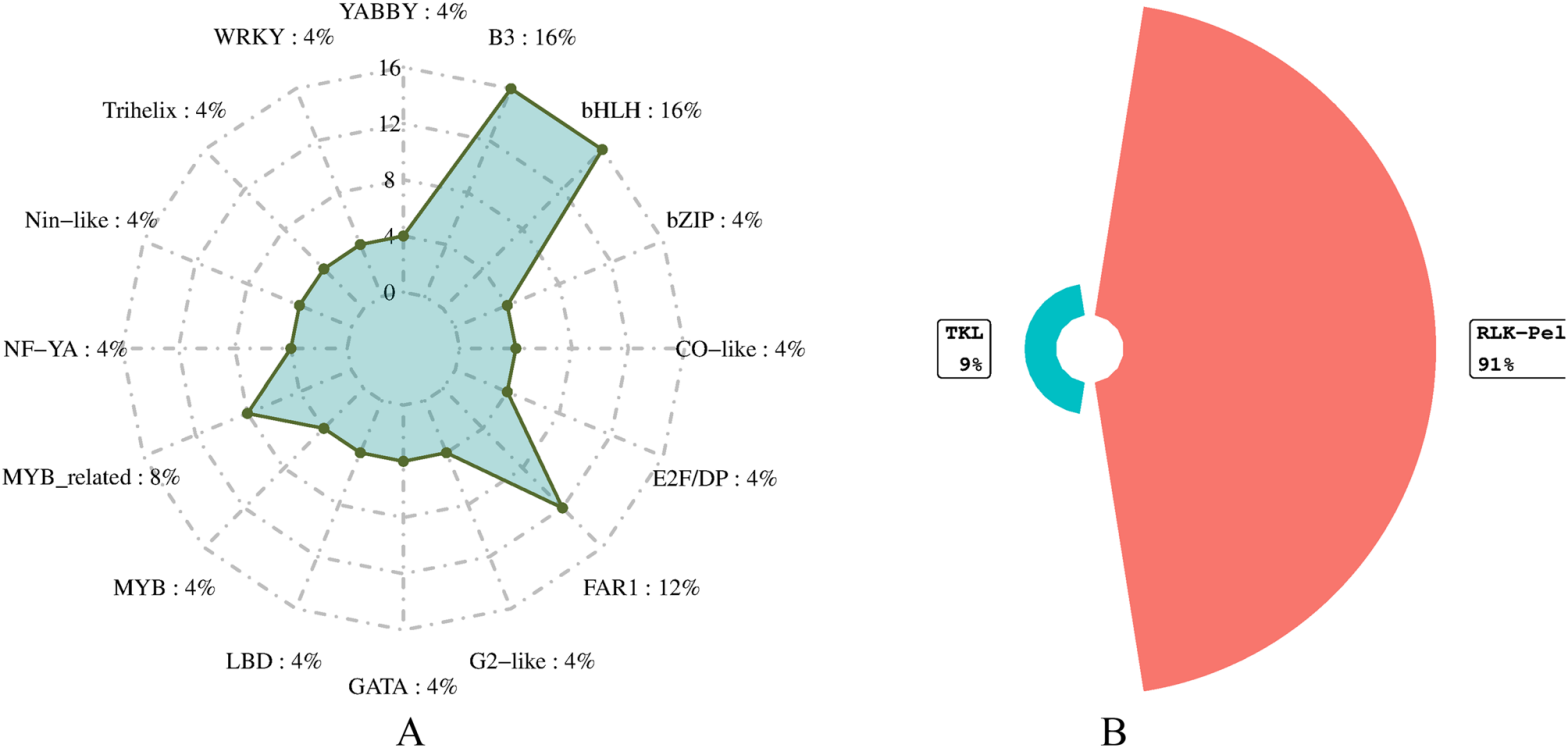
The family of transcription factors (spider chart: A) and protein kinases (circular barplot: B) associated with candidate genes.

In total, 16 TFs including B3, basic Helix-Loop-Helix (bHLH), basic leucine zipper (bZIP), CO-like, E2F/DP, FAR-RED IMPAIRED RESPONSE1 (FAR1), Golden 2-Like (G2-like), GATA, LATERAL ORGAN BOUNDARIES DOMAIN (LBD), myeloblastosis (MYB), MYB_related, NF-YA, Nin-like, Trihelix, WRKY and YABBY were identified. These TFs were encoding one to four genes (Fig. 4A). Furthermore, the tyrosine kinase-like (TKL) kinases are a group of serine-threonine protein kinases with sequence similarity to tyrosine kinases. The present study, *HORVU.MOREX.r3.4HG0332630* gene was related to TKL kinases (Fig. 4B).

### Protein kinases

A total, ten genes were related to receptor-like kinase (RLKs) family in this study (Table 4).

**Table 4:** The genes related to receptor like kinase family in barley.

Chr	Gene
2H	HORVU,MOREX.r3.2HG0136480
2H	HORVU,MOREX.r3.2HG0136510
2H	HORVU,MOREX.r3.2HG0136790
2H	HORVU,MOREX.r3.2HG0136830
2H	HORVU,MOREX.r3.2HG0136860
5H	HORVU,MOREX.r3.5HG0422540
5H	HORVU,MOREX.r3.5HG0425730
7H	HORVU,MOREX.r3.7HG0656210
7H	HORVU,MOREX.r3.7HG0656410
7H	HORVU,MOREX.r3.7HG0656900

### miRNAs and their target genes

**Figure 5:**
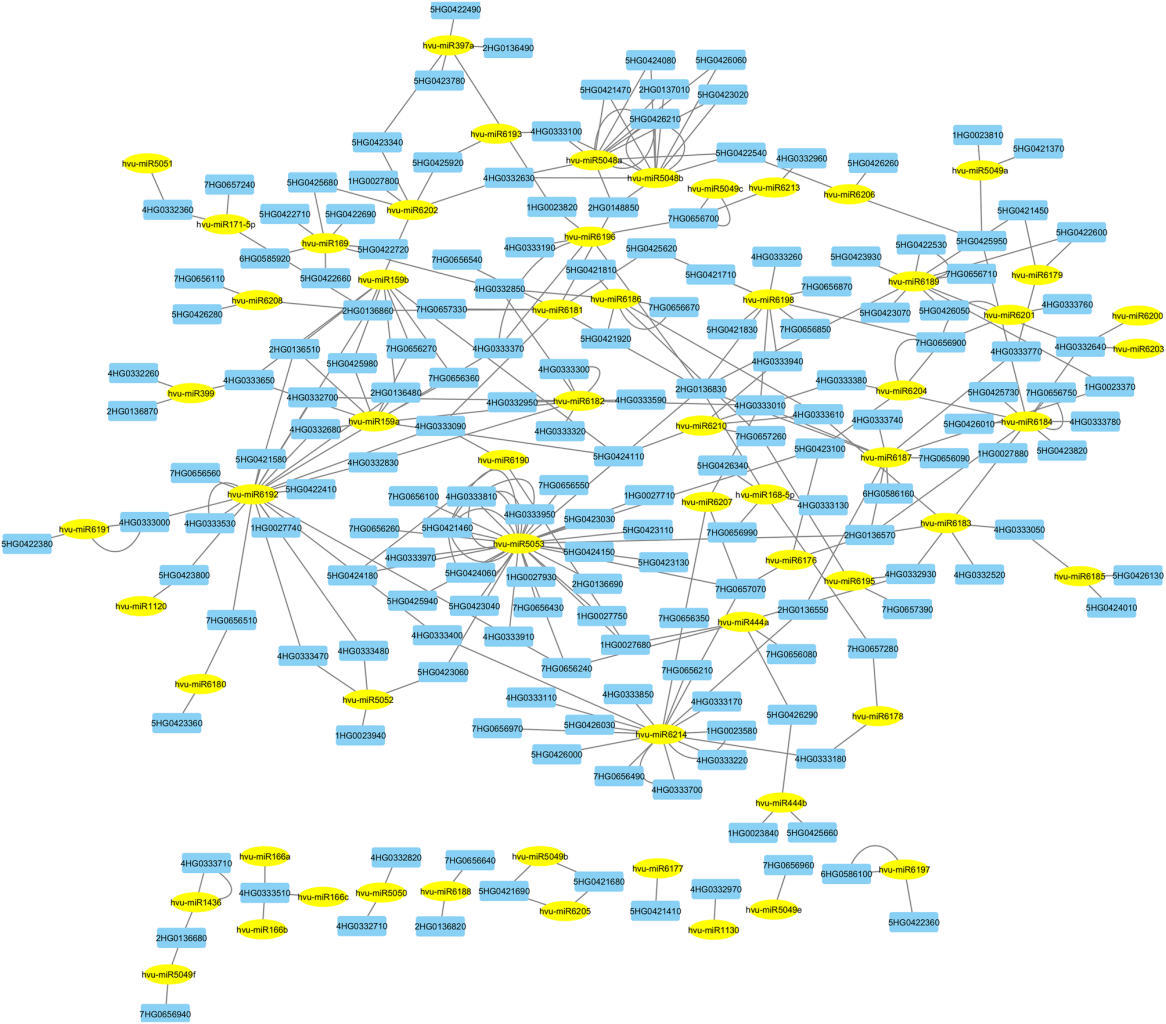
miRNA-target gene interaction network. Blue nodes represent target genes and yellow nodes showed miRNAs.

The miRNAs are small non-coding RNAs that play a crucial role in post-transcriptional gene regulation. They bind to complementary sequences in target mRNA transcripts, leading to their degradation or translational repression. The psRNATarget server is a tool for detecting target genes of miRNAs. It uses a scoring schema to analyze complementary matching between miRNA and mRNA sequences, enhancing the discovery of miRNA-mRNA interactions. This server predicted 60 miRNAs that regulate the 176 target genes among all identified candidate genes (Fig. 5). Most of miRNAs were targeted more than one gene. Moreover, the top interactive miRNAs including hvu-miR5053, hvu-miR6192 and hvu-miR6214 were associated with the most target genes. Most of miRNAs which targeted candidate genes were related to tolerance-stress.

## Discussion

In the present linkage map, the average marker distance was 1.45 cM. Lander and Botstein suggested that if the average distance of markers is less than 20 cM in the linkage map, it will be suitable for tracing QTLs^21^. According to this, the current linkage map is reliable to identify QTLs. So, the accuracy of the results is confirmed. However, it is suggested that for complementary studies, QTLs should also be tracked in other populations.

To confirm the QTL mapping findings, the results of the present study were compared with the reports of others. Under control condition, Moursi et al*.* identified one QTL for S/RL on chromosome 7H and seven QTLs for GP on chromosomes 1H, 2H, and 5H^15^. Moreover, under control condition, Sayed et al*.* mapped QTLs controlling GP and MGT on chromosomes 6H and 2H, respectively^22^. Makhtoum et al*.* identified two QTLs affecting RL on chromosome 7H at marker intervals of 66 and 134 cM under control condition^23^. Bálint et al*.* identified the QTLs for SL and RL on chromosome 1H (61.05 cM), QTL for SDW on chromosome 2H (88.26 cM), a QTL for SL on chromosome 7H (76.75 cM) and QTLs for SL, RL, SDW on chromosome 7H (82.60 cM) under control conditions^24^.

Moursi et al*.* identified a total of 21 QTLs for RL on chromosomes 1H, 2H, 3H, 4H and 7H, two QTLs for S/RL on chromosome 1H, and three QTLs for GP on chromosomes 1H, 2H and 6H under drought stress conditions^15^. In a study under drought conditions, two QTLs affecting RW were identified on chromosomes 1H (32 cM) and 2H (90 cM)^23^. Bálint et al*.* mapped the QTLs for SL, RL and SDW on chromosome 1H (61.05 cM), QTLs for SL and SDW on chromosome 2H (88.26 cM), a QTL for SL on chromosome 3H (163 cM), QTLs for SL and SDW on chromosome 5H (69.40 cM), QTLs for SL and SDW on chromosome 7H (76.75 cM) and QTLs for SL and SDW on chromosome 7H (82.60 cM) under drought stress conditions^24^. Arifuzzaman et al*.* identified five QTLs for SDW on chromosomes 2H (41.1, 81 and 98.21 cM), 5H (126.77 cM) and 6H (68.1 cM), three QTLs for RW on chromosomes 2H (41.1 cM), 3H (118.72 cM) and 5H (125.1 cM), seven QTLs for RDW on chromosomes 1H (39 and 123.09 cM), 2H (44.79 cM), 3H (118.72 cM), 4H (131 cM), 5H (126.77 cM) and 7H (42.5 cM), five QTLs for R/SW on chromosomes 1H (39 and 123.09 cM), 3H (118.72 cM), 5H (126.77 cM) and 7H (42.5 cM) under drought stress conditions^25^.

In a study under salinity stress, two QTLs on chromosomes 5H and 7H were identified in response to salinity stress^3^. Under salinity stress condition, Sayed et al*.* identified QTLs affecting RL and GP on chromosomes 2H and 5H, respectively. They also mapped QTLs related to SL on chromosomes 2H and 5H^22^. Furthermore, in another study under salinity stress, three QTLs associated with RL were identified on chromosomes 1H (126 cM), 2H (4 cM) and 4H (140 cM)^23^.

In summary, comparing the findings of the present study with the mentioned reports indicates the confirmation of some QTLs such as qSL-D-1H, since the was also identified in the report of Bálint et al*.*^24^. However, some QTLs have not been reported so far and are probably novel. In general, the differences in QTLs reported are related to factors such as differences in parents, population type, experimental accuracy, and levels of experimental treatments. Consequently, to confirm the findings, further experiments with different parents are needed.

Overall, the current study, a total of 14 major QTLs were tracked under different environmental conditions. The confirmed QTLs can be used in marker-assisted selection (MAS) projects. For this purpose, Flanking markers can be used to screen cultivars tolerant to drought and salinity stresses. Also, other advantages of MAS include gene pyramiding, prevention of undesirable gene transfer, selection of traits with low heritability, savings in conducting complex experiments in the field and laboratory, elimination of unreliable phenotypic evaluation related to field experiments due to environmental effects, selection of cultivars at the seedling stage and special experiments where phenotypic evaluation is not practical^26^.

The results represented that candidate genes mostly involved in biological process related to response to stimulus, signal transduction, and reproduction which are very important terms in germination. From a genetic viewpoint, the process of seed germination is influenced by complex interactions like plant hormone signal transductions. In a study, it was suggested that signal transduction is essential for seed germination in *Brassica napus*^27^. Moreover, it was reported that the signal transduction system is very important for asparagus bean in protecting itself from salinity^28^. Overall, drought and salinity stresses have bad influences on growth and germination of plant^29^. To respond these abiotic stresses, plants have regulatory systems specially through transmitting signals. One of the intracellular signal transduction pathways is through a pathway called the Ca^2^^+^ signal system that is responsible for further signal transduction^30^. As mentioned, two of most important biological processes associated with candidate genes were response to stimulus and cellular response to stimulus. Actually, external stimuli such as salinity or drought stresses can stimulate the plant. Concentration of Ca^2+^ as an important ion in signal transduction system can change transiently in cells. It starts cascade of the signal and send it to the inside the cell. These changes can be detected through calcium receptors and induce subsequent response processes^31^. Also, the above intracellular messengers target many protein kinases in cells. These protein kinases can phosphorylate intracellular proteins and regulate them, furthermore transmit information. As our findings were detected, most important molecular functions were “protein histidine kinase binding”, “protein kinase binding” and “kinase binding”. Therefore, these molecular functions which mediated by protein kinases can activate some biological processes such as “response to stimulus” and “cellular response to stimulus”. Furthermore, cascade of signal transduction, signaling and response to hormone start and plant can response to stresses like drought and salinity. Since these are intracellular processes and functions, and the process of transmission and signaling will be activated, the most significant cellular components that were identified in this study were “intracellular protein-containing complex” and “transferase complex”. Another significant molecular function was “GTPase activity”. Small GTPases mediate intracellular signal transduction. They are molecular switches between GDP and GTP state^32^. Therefore, in response to stimulus and stresses, this molecular function is activated to begin some signal transduction pathways which mediate through protein kinases. Totally, most terms of GO represented responses of the plant to stresses. In another study on the transcriptome of barley related to drought tolerance, one of the most important biological processes was regulation of signal transduction which includes significant mechanisms of environmental sensing^33^. It was reported that the first signal transduction reaction is controlled by phosphorelay pathway and involving phospholipases and protein kinases and rapid response to the stress^33^.

The PPI network showed that *HORVU.MOREX.r3.6HG0585670* (MLOC_70470.2) is one of genes in this network. This gene explains the E2F/DP family. E2F is a family of TFs implicated in the regulation of genes required for progression through G1 and entry into the S phase^34^. Another important gene in this network was *HORVU.MOREX.r3.5HG0426290* (MLOC_36395.1). In an experiment on the transcriptome of barley related to drought tolerance, MLOC_36395 gene was upregulated in one of the genotypes^33^.

In the current study, 14 TFs were identified in the pathway of resistance to drought and salinity stresses. The TFs are crucial regulatory proteins that play important roles in growth, development, and stress response^35^. Also, TFs activate the expression of stress-inducing genes and have significant effect on signal transduction pathways. They can help plants adjust to different environments. Moreover, TFs associated with candidate genes were identified that mostly belonged to B3 and bHLH family group with four encoding genes. Also, FAR1 family group had three encoding genes (Fig. 4A). Also, the B3 play an important role in seed maturation and specifically have been found in plants^36^. In another experiment on transcriptome investigation of barley under mild drought stress, B3 and bHLH families have prevailed in differentially expressed genes^37^. Additionally, the bHLH proteins form a large superfamily of transcriptional regulators which are involved in the regulation of the cell cycle and many developmental processes^38^. Also, bHLH TFs play significant regulatory roles in stress responses like salinity and drought stress^39^. Some bHLH TFs regulate growth and photosynthesis and thus bestow drought tolerance^40^. In addition, the important role of bHLH TFs in plant tolerance to salinity was reported^41^. FAR1 protein regulates ABA signaling in some plants and can contribute to response and tolerance to abiotic stresses. Moreover, this TF is important for plant growth and development as well as in adaptation and domestication^42^.

The protein kinases are regulatory proteins that act as master regulators in various biological processes^43^. The RLK/Pelle gene family regulates growth and developmental processes. In addition, this gene family interacts with symbionts and pathogens^44^. Overall, RLK gene family is involved in the response to biotic and abiotic stresses^45^. It was reported that RLK/Pelles and TKLs are components of signaling networks that regulate the activation of the defense response^46^.

The miRNAs have been involved in many areas of plant growth, like stress response and reproduction. The present study, most of miRNAs were associated with more than a target gene. For example, three miRNAs including hvu-miR5053, hvu-miR6192 and hvu-miR6214 were associated with 28, 21 and 16 target genes, respectively. In a study, hvu-miR6214 was predicted to target genes involved in photosynthesis pathway, cell division and hormone activity. Therefore, it is associated with plant development and growth^47^. The results showed that hvu-miR159a and hvu-miR159b were associated with some of target genes. In another study, hvu-miR159b was identified as a key regulator miRNA in drought condition in barley and its expression confirmed through quantitative real-time polymerase chain reaction (qPCR) method^48^. Also, hvu-miR159a was introduced as a key miRNA in germination and seeding growth of Tibetan hulless barley^49^. Variety number of salinity-induced miRNAs has been identified in plants. In Arabidopsis, the expression of miR159 was significantly induced in salinity stress^50^. Deng et al*.* identified miR159 as an important miRNA in barley under salinity stress. It has been showed that miR159 is involved in signal transduction that is a common survival strategy for plants to adjust to stress^51^. Additionally, the miR166a, miR166b and miR166c were associated with HORVU.MOREX.r3.4HG0333510. It was found that the expression pattern of miR166 family showed prominent roles in response to high salinity, drought and low temperature in soybean^52^. It was shown that three members of the miR166 family were expressed during embryogenesis in Arabidopsis^53^. Also, hvu-miR166a was introduced as a key regulatory miRNA between water and drought conditions and its expression was confirmed using real-time PCR^48^. Totally, most of miRNAs related to candidate genes were associated with response and tolerance to stresses.

## Conclusions

A total of 422 unique candidate genes were identified related to most major QTLs. Most of these candidate genes were active in signal transduction and response to stimulus biological processes. Also, the main molecular function of these candidate genes was histidine kinase binding and histidine phosphor-transfer kinase activity. Investigation of TFs and protein kinases that regulate the expression of candidate genes confirm the activation of cascade of tolerance and response to the stresses as well as development and growth pathways. In addition, miRNAs related to these candidate genes were mostly related to plant development, growth and response to stresses. The results represented that these candidate genes were the major players in response to drought and salinity stress conditions during the germination of barley. These findings can be used for understanding the molecular basis of barley germination under drought and salinity stress conditions and thus used in advanced breeding technologies of barley.

## Methods

### Plant materials

In order to identify closely linked QTLs associated with germination indices in response to control, drought and salinity conditions in barley, the three separate experiments were carried out in the botanical laboratory of Gonbad Kavous University. The experiments were conducted in a completely randomized design (CRD) with three replications. The plant materials included 103 recombinant lines (RILs) resulting from the crossing of Badia × Kavir during the germination stage. Kavir and Badia cultivars are six-row and are licensed by SPII (Seed and Plant Improvement Institute) and ICARDA (International Center for Agricultural Research in the Dry Areas), respectively. Also, Kavir and Badia cultivars are tolerant and sensitive to drought and salinity stresses, respectively^13^. This is because the tolerant Kavir cultivar had a significant difference (*P* < 0.01) with the sensitive Badia cultivar in terms of germination percentage, germination rate, radicle length, plumule length, radicle weight and plumule weight.

### Experimental treatments

First, one hundred healthy seeds from each of the lines were selected and sterilized with a 2% sodium hypochlorite solution for 10 min and then washed three times with distilled water. Also, the petri dish was sterilized in an autoclave (121 °C, 1.5 atm, 20 min). Finally, the seeds were transferred to a petri dish on sterile filter paper. The seeds were cultivated under control conditions using distilled water. However, for the treatment of seeds under drought stress conditions, 6.25 gr of polyethylene glycol (PEG) was used in 100 cc of distilled water (twice distilled). The amount of PEG for creating the necessary potential was calculated using the method of Michel & Kaufmann^54^. Also, for the treatment of seeds under salinity stress conditions, NaCl (12 ds/m) was used, and to calculate the necessary amounts, the method of van’'t Hoff was used^55^. Finally, daily counting of seeds under different conditions was performed for seven days. Overall, all the methods were performed in accordance with relevant guidelines and regulations.

### Evaluation of germination indices

A total of 10 seedlings from each of the lines were randomly sampled and germination indices were calculated. In order to ensure the normality of the phenotypic data, Pearson’s skewness and kurtosis tests were used. Also, other descriptive statistics including mean and range were estimated. All analyses of phenotypic data were calculated using SPSS software version 27.0.1. A total of 17 germination indices including GP, RL, SL, GI, GRI, SVI, MGT, SWVI, SLVI, RLI, RDWI, SLI, SDWI, R/SL, R/SDW, R/SLI and R/SDWI were calculated^16,56–60^.

### Linkage map development

The present linkage map was prepared based on the maps of Taliei et al.^12^ and Sabouri et al.^13^. Based on this, the polymorphism between the parents was done using Simple Sequence Repeat(SSR) markers, in addition several types of dominant markers including Inter Simple Sequence Repeat(ISSR), Expressed Sequence Tag (EST), Transposable Element (TE), Start Codon Target (SCoT), CAAT Box-Derived Polymorphism (CBDP), Inter-Retrotransposon Amplified Polymorphism (IRAP), Random Amplified Polymorphic DNA (RAPD), Intron–exon Splice Junctions (ISJ), inter Primer Binding Site (iPBS), combined iPBS-iPBS and combined ISSR-iPBS markers. Therefore, first, leaf samples were taken from the seedlings during the three-leaf stage, and then DNA was extracted from the leaves using CTAB method^61^. The quantity and quality of DNA samples were controlled by spectrophotometry and DNA horizontal electrophoresis (0.8% agarose gel), respectively. PCR was performed with a thermocycler (Bio-Rad, USA). The PCR reaction solution for SSR markers were contained 2.5 µl of DNA, 0.6 µl of dNTP (10 mM), 0.48 µl of MgCl_2_ (50 mM), 0.75 µl of forward primer (10 pmol), 0.75 µl of reverse primer (10 pmol), 1 µl of PCR buffer, 0.12 µl of *Taq* DNA polymerase (5 U/µl) and 3.8 µl of sterile water. Then, using DEPC water, the test solution was brought to a volume of 10 µl. Also, thermal cycles for PCR were programmed as touchdown. Finally, the PCR product was separated using Poly Acrylamide Gel Electrophoresis (PAGE) method and staining was performed using fast silver nitrate method (rapid silver staining)^62^. After extracting genetic data and preparing a genetic matrix, Mendelian ratios (1:1) were tested through chi-square under SPSS software version 27.0.1. Map Manager QTX software (https://gaow.github.io/genetic-analysis-software/m/map-manager-qtx/) was used to prepare the linkage map. Marker distances were calculated using Kosambi map function^63^.

### Genome-wide composite interval mapping (GCIM)

The closely linked QTLs were identified by GCIM method. To determine the LOD (logarithm of odds), a permutation test with 1000 reshuffling was used. Therefore, to identify closely linked QTLs were used R software QTL.gCIMapping.GUI v2.0 (https://pubmed.ncbi.nlm.nih.gov/31890145/). After tracking the closely linked QTLs, the international standard method was used to name them. The criterion for identifying major QTLs was the coefficient of determination values. Accordingly, QTLs with a coefficient of determination higher than 20% were identified as major QTLs.

### Detection of candidate genes associated with major QTLs

The genes underlying each major QTL were identified in the 2 Mb intervals on either side (upstream and downstream) of QTL’s peak position (total 4 Mb regions). This procedure was performed through the BioMart tool in Ensembl Plants (https://plants.ensembl.org/biomart/martview). A circular plot was drawn using TBtools software version 2.086 (https://github.com/CJ-Chen/TBtools-II) to visualize the position of each major QTL.

### GO analysis

The significant enrichment analysis (*P* < 0.05) of GO terms was performed through TBtools software version 2.086 (https://github.com/CJ-Chen/TBtools-II) on all candidate genes of major QTLs.

### PPI network construction

Cytoscape software version 3.10 (https://cytoscape.org) and STRING version 12 (https://string-db.org) web tool were used for investigating the PPI network of identified genes. Overall, cytoscape software was used for visualization of this gene network. This software relies on the correlation levels among the relevant genes. Moreover, background protein set information in STRING includes a comprehensive collection of protein–protein associations for various organisms. These associations are derived from high-throughput experimental data, database mining, literature analysis, and genomic context predictions. STRING integrates and ranks these associations, extending them to orthologous protein pairs in other organisms when applicable. STRING utilizes completely sequenced genomes and exhaustive ontology classifications to transfer interaction evidence between organisms, enabling PPI prediction across a wide range of organisms.

### Finding TFs and protein kinases associated with candidate genes

For identifying potential TFs, Plant-TFDB version 5.0 (http://planttfdb.gao-lab.org) was used. For detecting kinase coding gens associated with candidate genes, iTAK version 18.12 (http://itak.feilab.net/cgi-bin/itak/index.cgi) was used.

### Finding miRNAs related to candidate genes

The plant small RNA analysis server (psRNAtarget) (https://www.zhaolab.org/psRNATarget/home) was used to predict miRNA/target pairs from candidate genes associated with major QTLs. psRNAtarget evaluates sequence complementarity between miRNAs and target genes using a scoring system benchmarked. Cytoscape software version 3.10 (https://cytoscape.org) was used to create microRNA-target gene interaction network.

### Guidelines and legislation

The study was conducted in accordance with relevant guidelines and legislation.

### Supplementary Information

Supplementary Information.

## Data Availability

All data generated or analysed during this study are included in this published article and Supplementary file.
