# Supplementary material for: Detection of closely linked QTLs and candidate genes controlling germination indices in response to drought and salinity stresses in barley
Source: Sci Rep. 2024 Jul 8;14:15656. doi: 10.1038/s41598-024-66452-9 (PMC11231201; doi:10.1038/s41598-024-66452-9)
Supplement: Supplementary file 1 — Supplementary Information. [file 41598_2024_66452_MOESM1_ESM.docx]

***Supplementary Information***

**Detection of closely linked QTLs and candidate genes controlling germination indices in response to drought and salinity stresses in barley**

Hossein Sabouri^1*^, Zahra Pezeshkian^2,3^, Fakhtak Taliei^1^, Mahjoubeh Akbari^1^, Borzo Kazerani^4^

1. Department of Plant Production, College of Agriculture Science and Natural Resource, Gonbad Kavous University, Gonbad, Iran.
2. Department of Animal Sciences, Faculty of Agricultural Sciences, University of Guilan, Rasht, Iran.
3. BioGenTAC Inc., Technology incubator of Agricultural Biotechnology Research Institute of Iran-North Branch (ABRII), Rasht, Iran.
4. Department of Plant Breeding and Biotechnology, Faculty of Plant Production, Gorgan University of Agriculture Science and Natural Resources, Gorgan, Iran.

^*^ Correspondence: hossein.sabouri@gonbad.ac.ir

**Content**

| Supplementary Table 1 | …………………………………………………………………………………….. | 2 |
| --- | --- | --- |
| Supplementary Figure 1 | …………………………………………………………………………………….. | 3 |

**Supplementary Table 1.** Descriptive statistics of germination indices under control, drought and salinity conditions in barley.

| **Condition** | **Germination index** | **Rang** | **Mean** | **Skewness**  **(Pearson)** | **Kurtosis**  **(Pearson)** |
| --- | --- | --- | --- | --- | --- |
| Control | GP | 5 | 97.36 | 0.07 | -1.31 |
|  | SL | 6.3 | 11.45 | 0.11 | -0.24 |
|  | GI | 0.29 | 3.53 | -1.21 | 0.13 |
|  | SLVI | 157.5 | 283 | 0.17 | -0.21 |
|  | R/SL | 1.14 | 1.07 | 1.18 | 1.67 |
| Drought | RL | 8.5 | 9.43 | 0.27 | 0.29 |
|  | SL | 6 | 9.71 | -0.26 | -0.46 |
|  | GI | 1.43 | 2.88 | -0.37 | 0.13 |
|  | SWVI | 2.42 | 2.3 | 0.34 | -0.06 |
|  | SLVI | 136.5 | 195.6 | 0.01 | -0.79 |
|  | RDWI | 85.94 | 74.18 | 0.61 | 1.15 |
|  | SLI | 48.89 | 85.12 | -0.11 | 0.19 |
|  | SDWI | 55.17 | 71.87 | 0.33 | 0.18 |
|  | R/SL | 0.85 | 0.98 | 0.05 | 0.1 |
|  | R/SDW | 0.93 | 0.92 | 0.41 | 0.28 |
|  | R/SLI | 0.94 | 0.94 | 0.01 | -0.13 |
| Salinity | GP | 36 | 58.21 | 0.5 | 0.85 |
|  | RL | 7.5 | 6.24 | 0.28 | -0.21 |
|  | SL | 8.5 | 7.18 | 0.34 | 0.06 |
|  | GRI | 68 | 70.14 | 0.46 | 0.72 |
|  | SVI | 14.59 | 11.96 | 1.06 | 1.46 |
|  | MGT | 5.23 | 29.72 | 1.94 | 1.21 |
|  | SWVI | 2.41 | 1.17 | 0.96 | 1.6 |
|  | RLI | 60.62 | 52.38 | 0.11 | -0.71 |
|  | RDWI | 80.25 | 63.35 | 0.38 | -0.4 |
|  | SLI | 53.48 | 63.02 | -0.2 | -0.86 |
|  | R/SDW | 1.98 | 0.98 | 1.04 | 1.99 |
|  | R/SDWI | 1.67 | 1.05 | 1.21 | 1.14 |

GP (germination percentage), RL (root length), SL (shoot length), GI (germination index), GRI (germination rate index), SVI (seedling of vigor index), MGT (mean germination time), SWVI (seedling weight vigor index), SLVI (seedling length vigor index), RLI (root length index), RDWI (root dry weight index), SLI (shoot length index), SDWI (shoot dry weight index), R/SL (root/shoot ratio by length), R/SDW (root/shoot ratio by dry weight), R/SLI (root/shoot ratio by length index) and R/SDWI (root/shoot ratio by dry weight index).

**Supplementary Figure 1.** Linkage genetic map obtained from SSR, ISSR, EST, TE, SCoT, CBDP, IRAP, RAPD, ISJ, iPBS, iPBS-iPBS, and combined ISSR-iPBS markers in barley RILs caused Badia × Kavir cross.
